# The Chasm in Percutaneous Coronary Intervention and In-Hospital Mortality Rates Among Acute Myocardial Infarction Patients in Rural and Urban Hospitals in China: A Mediation Analysis

**DOI:** 10.3389/ijph.2022.1604846

**Published:** 2022-07-07

**Authors:** Miao Cai, Echu Liu, Peng Bai, Nan Zhang, Siyu Wang, Wei Li, Hualiang Lin, Xiaojun Lin

**Affiliations:** ^1^ School of Public Health, Sun Yat-sen University, Guangzhou, China; ^2^ College for Public Health and Social Justice, Saint Louis University, Saint Louis, MO, United States; ^3^ Department of Cardiovascular Surgery, Union Hospital, Tongji Medical College, Huazhong University of Science and Technology, Wuhan, China; ^4^ Department of Endocrinology, Union Hospital, Tongji Medical College, Huazhong University of Science and Technology, Wuhan, China; ^5^ Hubei Provincial Clinical Research Center for Diabetes and Metabolic Disorder, Wuhan, China; ^6^ Center for Genome Sciences and Systems Biology, School of Medicine, Washington University in St. Louis, Saint Louis, MO, United States; ^7^ Department of Data Science, Zhejiang University of Finance and Economics Dongfang College, Haining, China; ^8^ HEOA Group, West China School of Public Health and West China Fourth Hospital, Sichuan University, Chengdu, China; ^9^ Institute for Healthy Cities and West China Research Center for Rural Health Development, Sichuan University, Chengdu, China

**Keywords:** China, acute myocardial infarction, rural-urban disparity, percutaneous coronary intervention, mediating effect

## Abstract

**Objectives:** To determine to what extent the inequality in the ability to provide percutaneous coronary intervention (PCI) translates into outcomes for AMI patients in China.

**Methods:** We identified 82,677 patients who had primary diagnoses of AMI and were hospitalized in Shanxi Province, China, between 2013 and 2017. We applied logistic regressions with inverse probability weighting based on propensity scores and mediation analyses to examine the association of hospital rurality with in-hospital mortality and the potential mediating effects of PCI.

**Results:** In multivariate models where PCI was not adjusted for, rural hospitals were associated with a significantly higher risk of in-hospital mortality (odds ratio [OR]: 1.19, 95% confidence interval [CI]: 1.03–1.37). However, this association was nullified (OR: 0.94, 95% CI: 0.81–1.08) when PCI was included as a covariate. Mediation analyses revealed that PCI significantly mediated 132.3% (95% CI: 104.1–256.6%) of the effect of hospital rurality on in-hospital mortality. The direct effect of hospital rurality on in-hospital mortality was insignificant.

**Conclusion:** The results highlight the need to improve rural hospitals’ infrastructure and address the inequalities of treatments and outcomes in rural and urban hospitals.

## Introduction

Ischemic heart disease was the second leading cause of death and years of life lost in China in 2017 [1], and acute myocardial infarction (AMI) is the most severe manifestation. AMI cases in China are projected to increase substantially from eight million in 2010 to 23 million in 2030 [2], compared to an overall declining trend of AMI incidence and mortality in developed countries [3–7]. The modest or marked decline in developed countries attributes mainly to the popularity of revascularization procedures, pharmacological strategies [5–9], and population-level measures, such as smoking cessation and blood pressure control [3, 4].

AMI requires prompt and effective reperfusion and revascularization therapies, which are often provided in specialized medical departments. Percutaneous coronary intervention (PCI) is a safe and effective treatment that is superior to other thrombolytics for patients with ST-elevation myocardial infarction (STEMI), as recommended by several guidelines [10–12]. Early reperfusion using primary PCI reduces the level of ischemic injury, preserves cardiac function, and is associated with substantially lower mortality rates [10–12]. With the remarkable economic development of the last 30 years, a broad spectrum of hospitals in urban China can now provide these therapies [13, 14]. By contrast, rural hospitals are much less capable of administering these services, given the sparse population, lack of affordability, and limited hospital volume and clinical capacity [13, 15–17]. This rural-urban disparity in the ability to provide adequate and effective cardiovascular intervention treatments may have differentially affected AMI outcomes in China. Explaining the gap in outcomes and treatments of AMI in rural and urban hospitals can pinpoint a systematic weakness in the healthcare delivery system and meet the distinct need of geographically disadvantaged rural residents in China, affecting an estimated 555 million people [18].

Prior studies have reported nonsignificant disparity regarding the treatment and in-hospital mortality of AMI patients in rural and urban hospitals in China [15, 19]. However, these studies were limited by relatively small sample sizes and outdated data. Accordingly, we evaluated the disparity of treatments and in-hospital outcomes for AMI patients hospitalized in rural and urban hospitals and identified the potential mediators by leveraging the breadth of an extensive province-wide hospital discharge data in China.

## Methods

### Data Source

We used the hospital discharge data of patients hospitalized in 162 tertiary and secondary hospitals in Shanxi Province, China, from 01 January 2013, to 31 December 2017 [20–24]. This database was used to manage all the hospitalized patients in the region and was standardized in 2011 by the former Ministry of Health, China. Patient demographic information (age and gender), socioeconomic status (marital status and occupation), one primary diagnosis and up to 10 secondary diagnosis codes (International Classification of Diseases, Tenth Revision, Clinical Modification [ICD-10-CM]), up to seven medical procedure codes (International Classification of Diseases, Ninth Revision, Procedure Coding System [ICD-9-PCS]), disease severity at admission, and discharge status were provided to the research team. All unique patient identifiers such as patient names and identification numbers were excluded before accessing the data. The Institutional Review Board of Sichuan University approved the study (approval number: K2020007).

### Study Sample

We selected patients whose primary ICD-10-CM diagnosis code contained “I21” during their hospitalization between 01 January 2013, and 31 December 2017 (N = 82,884). We further excluded the patients below the age of 18 at admission and those with unknown age and sex, yielding a final sample size of 82,677 patients. The final sample included 14,340 (17.3%) patients from 90 rural hospitals and 68,337 (82.7%) patients from 68 urban hospitals.

### Rurality of Hospitals

According to the rural-urban area code in the classification codebook published by the National Bureau of Statistics [25], the sample hospitals were classified as rural or urban hospitals. Hospitals located in the areas with the area codes 111 and 121, which indicates a central urban district and center areas of a town, were defined as urban hospitals. In contrast, the hospitals with other area codes (112, 122, 123, 210, and 220) were defined as rural hospitals. The details of the rural-urban area classification codes and associated meanings are provided in Supplementary Table S1.

### Outcomes

The primary outcome in this study was in-hospital mortality, defined as all causes of death during hospitalization. Since in-hospital mortality only captures the outcome during hospitalization and the data following discharge is unavailable, we further used non-recovery as the outcome to evaluate the robustness of our results in sensitivity analyses [23].

### Percutaneous Coronary Intervention

PCI is a non-surgical procedure that uses a catheter to treat the stenotic coronary artery. It has become the guideline-recommended and preferred treatment for most AMI over the past decades [8, 9, 25, 26]. In this study, PCI was identified using ICD-9-PCS procedure code during hospitalization (PCI: 36.00, 36.01, 36.02, 36.05, 36.06, 36.07, 36.09, and 00.66) [27]. Coronary artery bypass grafting (CABG) was not included as a covariate in this study because the CABG rate is low (0.9% in urban hospitals and less than 0.1% in rural hospitals).

### Covariates

**FIGURE 1:**
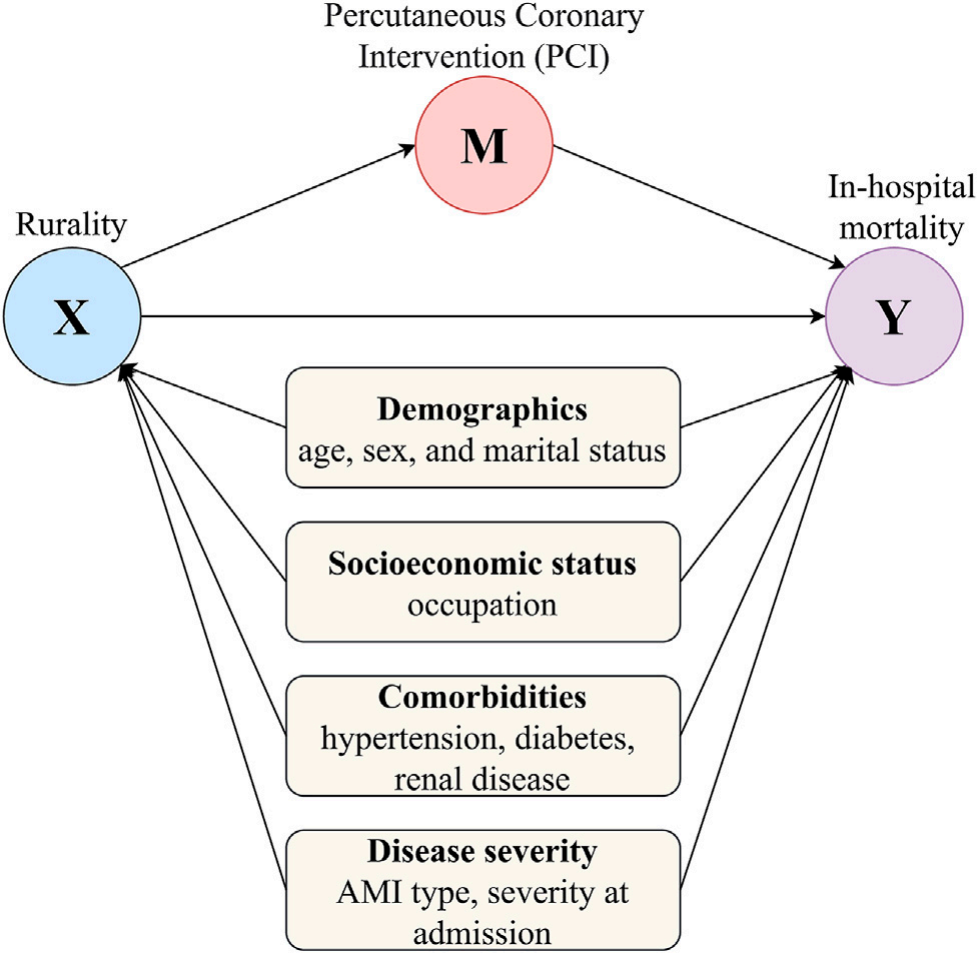
Directed acyclic graph for the mediating pathway of the association of hospital rurality with in-hospital mortality for acute myocardial infarction (Shanxi, China. 2013–2017). (X) represents the main exposure or treatment, the location of the hospital (rural or urban hospitals); (Y) is the outcome variable, the in-hospital mortality occurred to the acute myocardial infarction patients; (M) is the mediator variable, whether the patient had a percutaneous coronary intervention or not. Variable sets (demographics, socioeconomic status, comorbidities, and disease severity) in light yellow rounded rectangles are confounders.

Covariates were selected based on prior literature on the potential confounders of the association between rurality and in-hospital AMI mortality [28–30], data availability, and a directed acyclic graph (DAG) in Figure 1. Demographics and socioeconomic status included age, gender, marital status (married, unmarried, widowed, divorced, and other), and occupation (public institution, private institution, farmer, jobless, retired, and others). We selected three comorbidities, including hypertension, diabetes mellitus, and renal diseases, defined using ICD-10-CM diagnoses codes [27, 31–33]. AMI was categorized into three subtypes using ICD-10-CM codes: ST-elevation myocardial infarction, non-ST-elevation myocardial infarction (non-STEMI), and non-specified [28].

### Statistical Analysis

The characteristics of the overall sample and rural and urban hospitals are reported. We assessed the association between hospital rurality and in-hospital mortality in the overall sample using logistic regression models and different predictor variables. Since patients’ characteristics in rural and urban hospitals are different, we applied an inverse probability weighting (IPW) based on propensity scores to the sample, which resulted in a weighted pseudo sample where the characteristics of the patients in rural and urban hospitals are comparable [34–36]. We then conducted a logistic regression model on the weighted pseudo sample and estimated the association between hospital rurality and in-hospital mortality. The same set of models was then applied to the three subgroups of AMI patients (STEMI, non-STEMI, and non-specified) to examine the consistency of results from the overall sample. These logistic regression models reported odds ratios (OR) and associated 95% confidence intervals (CI).

We applied mediation analysis to test the hypothesis that the association between hospital rurality and in-hospital mortality is mediated by PCI (Figure 1). The difference method and the product method mentioned by VanderWeele [37] are not used in this study because they require two assumptions: 1) both the mediator and outcome models are linear regressions (this assumption is violated because both the mediator and outcome are binary variables and logistic regressions were applied) and 2) the mediator and treatment enter the model additive and without interactions (this requires additional assumption that cannot be verified empirically). Instead, we used a more general mediation analysis approach, proposed by Imai et al. [38], that can accommodate a wide range of statistical models, including logistic regressions, and requires no assumption on the additive effect or interactions. The total effect, average causal mediation effect (ACME), average direct effect (ADE), and proportion mediated were reported. The ACME, also known as mediated effect or indirect effect, is the effect of the treatment on the outcome that works through the mediator [39]. We estimated the percentage of mediation and the statistical significance using the mediation package in R [37, 40–42]. The portion of mediation is the ACME size relative to the main treatment’s total effect on the outcome. A proportion greater than 100% indicates that the size of the mediated effect is greater than the total effect, and this may happen if the directions of the total effect and the mediated effect are opposite. Bias-corrected and accelerated (BCa) 95% CIs of mediation effects were estimated using nonparametric bootstraps with 1000 resamples [42]. Similar to the logistic regression models, we applied mediation analyses to the three subgroups of AMI patients to check the results’ consistency as sensitivity analyses.

Due to the Chinese culture of filial piety, strong family ties, and financial affordability, patients may choose to withdraw from treatment at the terminal status and die in their homes [13]. Thus, using in-hospital mortality may not fully capture the patients’ actual outcomes, and estimated results may be subject to loss of follow-up bias. Therefore, we used non-recovery as the outcome and re-estimated the logistic regression models and mediation results in sensitivity analyses [23].

The data management, modeling, and visualization were conducted in statistical computing environment R 4.0.4. All statistical tests were two-sided, and a *p*-value<0.05 or a 95% CI excluded unity was considered statistically significant.

## Results

### Characteristics of the Overall Sample of Acute Myocardial Infarction Patients and by Rural and Urban Hospitals

Table 1 shows the demographic, socioeconomic, and health characteristics of the overall sample of AMI patients (N = 82,677) and by rural (N = 14,340, 17.3%) and urban (N = 68,337, 82.7%) hospitals. The overall AMI in-hospital mortality rate was 1.7%, and the crude mortality rate of rural hospitals was slightly but significantly higher than that of urban hospitals (1.9% vs. 1.7%, *p* = 0.046). Compared with rural hospitals, urban hospitals were more likely to admit a higher percentage of patients who were younger, male, married, and had an official job (worked in public and private institutions), as well as those who had more comorbidities (hypertension, diabetes mellitus, and renal disease). It is worth noting that the adoption of PCI procedures is significantly higher in urban hospitals (43.0%) than in their rural counterparts (13.4%).

**TABLE 1:** Characteristics of the acute myocardial infarction patients overall and by hospital rurality (Shanxi, China. 2013–2017).

Characteristics	Overall (N = 82,677)	Urban Hospitals (*n* = 68,337)	Rural Hospitals (*n* = 14,340)	*p*-value
Death	1409 (1.7)	1136 (1.7)	273 (1.9)	0.046
**Surgery interventions**				
PCI	31289 (37.8)	29369 (43.0)	1920 (13.4)	<0.001
**Socio-demographic variables**				
Age, years	61.9 (12.8)	61.8 (12.8)	62.8 (12.6)	<0.001
Female	20982 (25.4)	17028 (24.9)	3954 (27.6)	<0.001
Marital status				<0.001
Married	76165 (92.1)	63081 (92.3)	13084 (91.2)	
Unmarried	1599 (1.9)	1288 (1.9)	311 (2.2)	
Widowed	2672 (3.2)	2196 (3.2)	476 (3.3)	
Divorced	1201 (1.5)	1057 (1.5)	144 (1.0)	
Other	1040 (1.3)	715 (1.0)	325 (2.3)	
Occupation				<0.001
Public institution	5759 (7.0)	5187 (7.6)	572 (4.0)	
Private institution	11071 (13.4)	10246 (15.0)	825 (5.8)	
Farmer	39376 (47.6)	30019 (43.9)	9357 (65.3)	
Jobless	3353 (4.1)	3045 (4.5)	308 (2.1)	
Retired	15263 (18.5)	14077 (20.6)	1186 (8.3)	
Other	7855 (9.5)	5763 (8.4)	2092 (14.6)	
**Disease severity and comorbidities**				
AMI type				<0.001
STEMI	43944 (53.2)	35314 (51.7)	8630 (60.2)	
Non-STEMI	18797 (22.7)	17372 (25.4)	1425 (9.9)	
Not specified	19936 (24.1)	15651 (22.9)	4285 (29.9)	
Severity				<0.001
Normal	43515 (52.6)	34376 (50.3)	9139 (63.7)	
Emergent	21671 (26.2)	19085 (27.9)	2586 (18.0)	
Dangerous	17491 (21.2)	14876 (21.8)	2615 (18.2)	
Hypertension	39732 (48.1)	33521 (49.1)	6211 (43.3)	<0.001
Diabetes mellitus	16559 (20.0)	14325 (21.0)	2234 (15.6)	<0.001
Renal disease	1435 (1.7)	1247 (1.8)	188 (1.3)	<0.001

AMI, acute myocardial infarction; STEMI, ST-elevation myocardial infarction; PCI, percutaneous coronary intervention.

**FIGURE 2:**
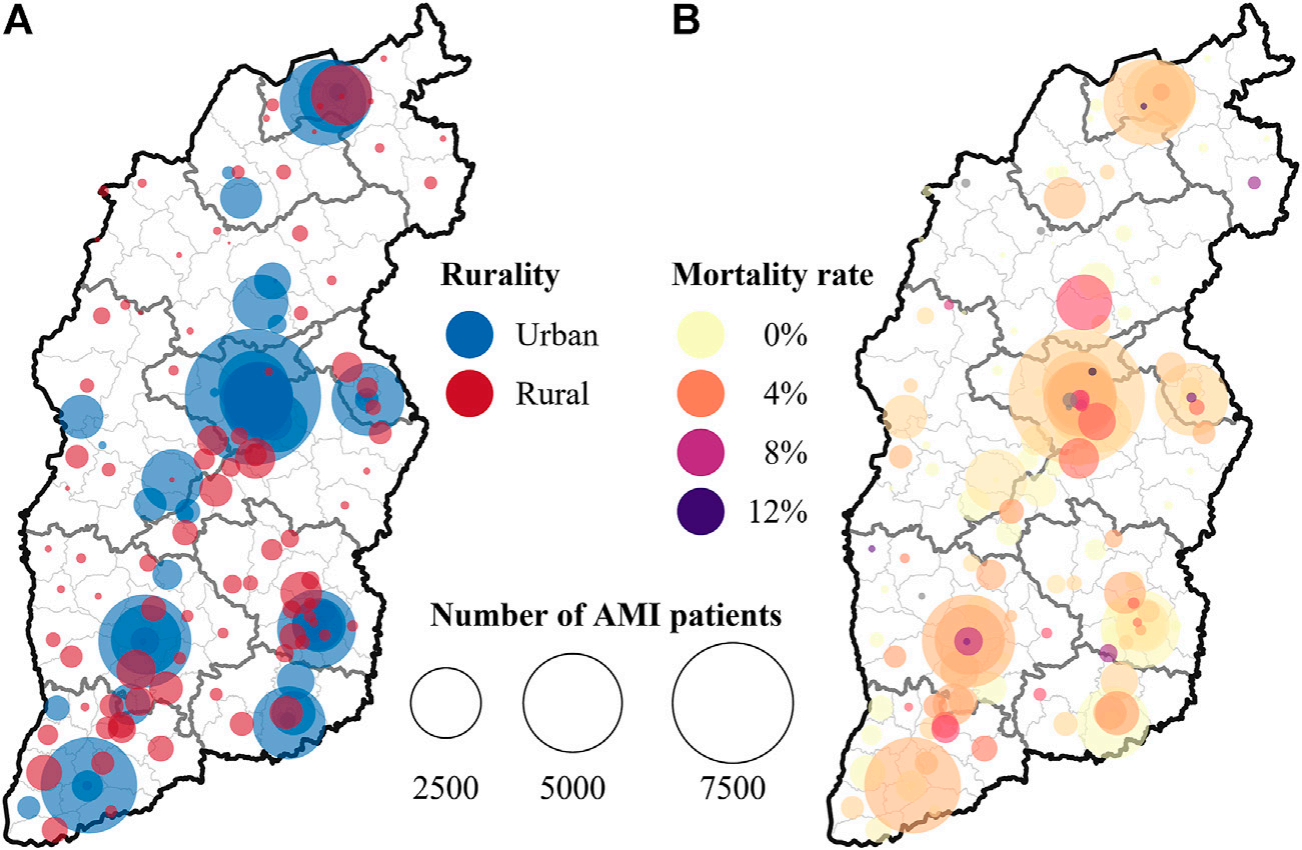
**(A)** number of acute myocardial infarction patients in rural and urban hospitals, and **(B)** in-hospital mortality rates for acute myocardial infarction in different hospitals (Shanxi, China. 2013–2017).

Figure 2 presents the geographical distribution of the sample rural and urban hospitals, with the dots’ size representing the number of AMI patients in each hospital during the study period. Urban hospitals tended to be medium- to large-volume hospitals, while rural hospitals were small- to medium-sized hospitals (Figure 2A). Figure 2B demonstrates that the AMI mortality rates tend to be higher in smaller hospitals.

### Association Between Hospital Rurality and Acute Myocardial Infarction Outcomes

We examined the disparity of AMI in-hospital mortality in rural and urban hospitals using logistic regression models with a different set of variable and adjustment methods (Table 2). In the crude model, rural hospitals had a significantly higher risk of in-hospital mortality (OR: 1.15, 95% CI: 1.01–1.31), and this disparity still held when we further adjusted for a minimally sufficient set of socioeconomic characteristics and disease severity specified in Figure 1 (OR: 1.19, 95% CI: 1.03–1.37). However, when we included PCI as a binary variable, the disparity of AMI mortality in rural and urban hospitals disappeared in the fully adjusted model (OR: 0.94, 95% CI: 0.81–1.08), and this null significance still held (OR: 0.90, 95% CI: 0.82–1.00) when we applied IPW to balance the distribution of covariates between rural and urban patients. In sensitivity analyses where the outcome of in-hospital mortality was replaced by non-recovery, we observed a similar pattern that including PCI as a predictor variable attenuated the association between rural hospitals and the risk of non-recovery (Supplementary Table S2).

**TABLE 2:** Association between hospital rurality and in-hospital mortality for acute myocardial infarction using different adjustment methods (Shanxi, China. 2013–2017).

Predictor variables	Unadjusted	Minimally sufficient set	Full	IPW
Rural	1.15 (1.01, 1.31)	1.19 (1.03, 1.37)	0.94 (0.81, 1.08)	0.90 (0.82, 1.00)
Age in 10 years		1.78 (1.69, 1.87)	1.60 (1.52, 1.69)	1.63 (1.57, 1.69)
Female		1.36 (1.20, 1.53)	1.31 (1.16, 1.48)	1.28 (1.17, 1.39)
Marital status (reference = married)				
Unmarried		0.80 (0.47, 1.28)	0.80 (0.46, 1.28)	0.75 (0.52, 1.05)
Widowed		1.09 (0.87, 1.35)	1.01 (0.81, 1.25)	1.19 (1.02, 1.38)
Divorced		1.16 (0.83, 1.57)	1.12 (0.80, 1.52)	0.82 (0.60, 1.09)
Other		1.44 (0.93, 2.12)	1.30 (0.84, 1.91)	1.31 (0.98, 1.72)
Occupation (reference = public institution)				
Private institution		0.89 (0.65, 1.25)	0.96 (0.70, 1.35)	1.36 (1.09, 1.71)
Farmer		0.78 (0.59, 1.06)	0.74 (0.55, 1.00)	0.87 (0.71, 1.07)
Jobless		0.84 (0.57, 1.23)	0.80 (0.54, 1.17)	0.80 (0.61, 1.06)
Retired		1.52 (1.14, 2.07)	1.52 (1.14, 2.07)	1.49 (1.22, 1.86)
Other		0.82 (0.59, 1.15)	0.72 (0.52, 1.02)	0.84 (0.66, 1.06)
Severity upon admission (reference = normal)				
Emergent		0.85 (0.73, 0.99)	0.88 (0.75, 1.02)	0.93 (0.84, 1.03)
Dangerous		1.99 (1.76, 2.25)	2.08 (1.84, 2.35)	1.82 (1.67, 1.99)
Hypertension		0.79 (0.71, 0.88)	0.80 (0.71, 0.89)	0.67 (0.62, 0.73)
Diabetes mellitus		1.11 (0.98, 1.26)	1.12 (0.99, 1.28)	1.07 (0.97, 1.17)
Renal disease		2.25 (1.76, 2.85)	1.93 (1.50, 2.44)	3.37 (2.90, 3.89)
AMI type (reference = STEMI)				
Non-STEMI		0.63 (0.54, 0.74)	0.55 (0.47, 0.63)	0.56 (0.50, 0.63)
Non-specified		1.33 (1.18, 1.50)	1.22 (1.08, 1.37)	1.04 (0.95, 1.13)
PCI			0.20 (0.16, 0.24)	0.22 (0.19, 0.25)

Effect estimates are presented as odds ratios with 95% confidence intervals. The minimally sufficient set is selected based on the directed acyclic graph in Figure 1. IPW, inverse probability weighting; AMI, acute myocardial infarction; STEMI, ST-elevation myocardial infarction; PCI, percutaneous coronary intervention.

Since the manifestation, disease severity, and treatment strategy varied across different AMI subtypes, we further estimated the rural-urban disparity of AMI outcomes in subtypes of AMI (STEMI, non-STEMI, and non-specified) separately, as shown in Supplementary Table S3. In the STEMI subgroup, we found a similar trend: rural hospitals showed a significantly higher risk of in-hospital mortality in the partially adjusted model (OR: 1.47, 95% CI: 1.22–1.76), but the significant association disappeared once PCI was included as a predictor variable in either the fully- or IPW-adjusted models. In non-STEMI and non-specified patients, the rural variable was a risk factor (although insignificant) in the partially adjusted models. Including PCI as a covariate in the fully- and IPW-adjusted models attenuated the effect sizes of rurality. In sensitivity analyses where non-recovery was used as the outcome (Supplementary Table S4), we observed consistent results that including PCI as a covariate reduced the magnitude of association between hospital rurality and patient non-recovery in the three subgroups of AMI.

### Acute Myocardial Infarction Patients in Rural Hospitals had Significantly Lower Use of Percutaneous Coronary Intervention

Table 3 presents the results of logistic regression models that examine the relationship between hospital rurality and PCI use, adjusting for the selected covariates in the overall sample and by AMI subtypes (STEMI, non-STEMI, and nonspecific types of AMI). In the overall sample of AMI patients, patients in rural hospitals had a significantly lower probability of PCI use (OR: 0.20, 95% CI: 0.19–0.22) compared to those in urban hospitals after adjusting for the covariates. When stratifying the sample by AMI type, we can still observe a consistently lower probability of PCI use in rural hospitals among patients with STEMI (OR: 0.24, 95% CI: 0.23–0.26), non-STEMI (OR: 0.27, 95% CI: 0.22–0.31), and nonspecific AMI (OR: 0.11, 95% CI: 0.10–0.13).

**TABLE 3:** Association between hospital rurality and the use of percutaneous coronary intervention in overall sample and subgroups of acute myocardial infarction patients (Shanxi, China. 2013–2017).

Predictor variables	Overall (N = 82,677)	STEMI (*n* =43,944)	Non-STEMI (*n* =18,797)	Non-specified (*n* =19,936)
Rural	0.20 (0.19, 0.22)	0.24 (0.23, 0.26)	0.27 (0.22, 0.31)	0.11 (0.10, 0.13)
Age in 10 years	0.74 (0.73, 0.75)	0.77 (0.76, 0.79)	0.67 (0.65, 0.69)	0.74 (0.72, 0.76)
Female	0.77 (0.74, 0.80)	0.77 (0.73, 0.82)	0.79 (0.73, 0.85)	0.72 (0.66, 0.78)
Marital status (reference = married)				
Unmarried	0.79 (0.70, 0.88)	0.82 (0.71, 0.95)	0.82 (0.63, 1.05)	0.69 (0.53, 0.90)
Widowed	0.57 (0.51, 0.63)	0.65 (0.57, 0.74)	0.44 (0.35, 0.55)	0.52 (0.40, 0.65)
Divorced	0.64 (0.56, 0.73)	0.67 (0.55, 0.80)	0.52 (0.38, 0.70)	0.76 (0.56, 1.01)
Other	0.55 (0.47, 0.64)	0.54 (0.43, 0.67)	0.52 (0.37, 0.71)	0.58 (0.40, 0.81)
Occupation (reference = public institution)				
Private institution	1.09 (1.02, 1.17)	1.19 (1.09, 1.31)	0.89 (0.77, 1.02)	1.12 (0.98, 1.29)
Farmer	0.82 (0.77, 0.87)	0.78 (0.72, 0.84)	0.89 (0.78, 1.01)	0.94 (0.83, 1.06)
Jobless	0.75 (0.69, 0.83)	0.79 (0.70, 0.90)	0.64 (0.52, 0.78)	0.84 (0.69, 1.02)
Retired	0.98 (0.91, 1.05)	1.07 (0.98, 1.18)	0.84 (0.73, 0.96)	1.01 (0.88, 1.16)
Other	0.59 (0.55, 0.64)	0.58 (0.53, 0.65)	0.73 (0.62, 0.86)	0.49 (0.41, 0.58)
Severity upon admission (reference = normal)				
Emergent	1.10 (1.06, 1.14)	1.17 (1.12, 1.23)	0.86 (0.80, 0.93)	1.22 (1.12, 1.32)
Dangerous	1.15 (1.11, 1.20)	1.24 (1.17, 1.30)	0.89 (0.82, 0.98)	1.19 (1.10, 1.29)
Hypertension	1.11 (1.08, 1.14)	1.13 (1.08, 1.17)	1.10 (1.03, 1.17)	1.06 (0.99, 1.13)
Diabetes mellitus	1.12 (1.07, 1.16)	1.14 (1.09, 1.21)	1.10 (1.02, 1.18)	1.07 (0.99, 1.16)
Renal disease	0.28 (0.24, 0.33)	0.33 (0.26, 0.40)	0.23 (0.16, 0.32)	0.24 (0.16, 0.34)
AMI type (reference = STEMI)				
Non-STEMI	0.60 (0.58, 0.63)	—	—	—
Non-specified	0.69 (0.67, 0.72)	—	—	—

Effect estimates are presented as odds ratios with 95% confidence intervals. STEMI, ST-elevation myocardial infarction; PCI, percutaneous coronary intervention.

### The Disparity of Acute Myocardial Infarction In-Hospital Mortality in Rural and Urban Hospitals is Mediated by Percutaneous Coronary Intervention Use

Since the logistic regression models revealed that including PCI as a covariate weakened the association between hospital rurality and AMI outcomes, we further tested the assumption that PCI is a mediator on the causal pathway of hospital rurality to AMI in-hospital mortality (Figure 1). Table 4 presents mediation analysis results in the overall sample and different subtypes of AMI. The total effect was the effect of rurality on in-hospital mortality, adjusting for the covariates but not the mediator PCI. The ADE is the effect of rurality on in-hospital mortality adjusting for the covariates and the mediator PCI. The ACME, also known as the mediated effect or indirect effect, was the effect of rurality on in-hospital mortality that worked through the mediator PCI, adjusting for the covariates. In the overall sample, PCI significantly mediated 132.3% (95% CI: 104.1–256.6%, *p*-value: 0.006) of the effect of hospital rurality on in-hospital mortality, and the average direct effect of rurality on in-hospital mortality became nonsignificant (*p*-value: 0.358) after accounting for PCI as a mediator. Sensitivity analyses using non-recovery as the outcome revealed a consistent result, although the average direct effect was statistically significant (Supplementary Table S5).

**TABLE 4:** Percutaneous coronary intervention mediating the effect of hospital rurality on in-hospital mortality in overall sample and subgroups of acute myocardial infarction patients (Shanxi, China. 2013–2017).

Sample	Overall (N = 82,677)		STEMI (*n* = 43,944)		Non-STEMI (*n* =18,797)		Non-specified (*n* =19,936)	
Odd ratio estimates	Estimate (95% CI)	*p*-value	Estimate (95% CI)	*p*-value	Estimate (95% CI)	*p*-value	Estimate (95% CI)	*p*-value
Total effect	1.003 (1.001, 1.01)	0.006	1.007 (1.004, 1.01)	<0.001	1.001 (0.995, 1.01)	0.71	0.997 (0.992, 1.002)	0.26
Average causal mediation effect (ACME)	1.004 (1.004, 1.005)	<0.001	1.005 (1.004, 1.01)	<0.001	1.002 (1.001, 1)	<0.001	1.006 (1.005, 1.01)	<0.001
Average direct effect (ADE)	0.999 (0.997, 1.012)	0.358	1.002 (0.999, 1.01)	0.13	0.999 (0.994, 1.01)	0.87	0.991 (0.986, 1)	<0.001
Proportion estimates								
Proportion mediated	132.3% (104.1%, 256.6%)	0.006	65.3% (49.7%, 160.0%)	<0.001	136.4% (−318.0%, 204.0%)	0.71	−20.6% (−167.9%, 110.0%)	0.26

STEMI, ST-elevation myocardial infarction; PCI, percutaneous coronary intervention; CI, confidence interval.

In subgroup analyses for different types of AMI (Table 4), we can see similar results in the STEMI subgroup, where PCI significantly mediates 65.3% (95% CI: 49.7–160.0%, *p*-value < 0.001) of the effect, and the average direct effect was nullified (*p*-value: 0.13) after including PCI as a mediator. In the non-STEMI and non-specified AMI group, PCI was not a significant mediator for hospital rurality and in-hospital mortality. In sensitivity analyses using non-recovery as the outcome (Supplementary Table S5), the mediator effect of PCI remained consistent. At the same time, PCI was still a significant mediator for non-STEMI and non-specified AMI subgroups.

## Discussion

Using a province-wide large sample of 82,677 AMI patients, we examined the association between hospital rurality and in-hospital mortality and the mediating effect of PCI on this association. When only demographics, socioeconomic status, and disease severity were controlled, we found that rural hospitals were associated with a significantly higher risk of in-hospital mortality (OR: 1.19, 95% CI: 1.03–1.37). However, this significant association was nullified when PCI was included in the model (OR: 0.94, 95% CI: 0.81–1.08). Further mediation analyses suggested that 132.3% (95%CI: 104.1–256.6%) of the positive effect of hospital rurality on in-hospital mortality was mediated by PCI, and the direct effect of hospital rurality on in-hospital mortality became insignificant when the PCI mediating effect was accounted for. This pattern was consistent when the outcome variable in-hospital mortality was replaced with non-recovery in our sensitivity analyses. Similar results were found in the subgroup of STEMI patients but not for non-STEMI or non-specified AMI patients.

Although treatment and quality of care have been improved for AMI patients over the last decades in China [13, 16], our study lays bare the deeply rooted rural-urban inequality in AMI treatment and outcomes. In our sample, 60.2% of the patients hospitalized in rural hospitals had STEMI, compared to a lower rate of 51.7% of STEMI patients in urban hospitals. However, PCI, the standard and preferred reperfusion therapy for applicable STEMI patients [8, 9], was only performed on 13.4% of the patients hospitalized in rural hospitals, compared to a substantially higher rate of 43.0% in urban hospitals in the same period. In our regression models, AMI patients cared for in rural hospitals had significantly higher in-hospital mortality rates (OR: 1.19, 95% CI: 1.03–1.37) compared to those in urban hospitals, even after controlling for demographics, socioeconomic status, disease severity, and comorbidities, which is consistent with previous findings [43–45].

More importantly, our study reveals that this rural-urban inequality of AMI outcomes is largely effaceable by enhancing rural hospitals’ capability to provide care that adheres to AMI treatment guidelines. Our regression models that account for PCI suggest that if the adoption rate of PCI procedures in rural hospitals were as high as that in urban hospitals, the inequality of in-hospital mortality between rural and urban hospitals would be eliminated (OR: 0.94, 95% CI: 0.81–1.08), especially for STEMI patients (OR: 1.15, 95% CI: 0.96–1.39), for whom PCI is a prioritized safe and effective therapy recommended by most guidelines [10–12]. The mediation analysis results demonstrate that 132.3% (95% CI: 104.1–256.6%) of the effect of hospital rurality on in-hospital mortality is mediated by PCI, with the direct effect becoming nonsignificant (*p*-value: 0.358) after including PCI as a mediator. This mediating effect is again consistent and significant in STEMI patients, for whom PCI is the recommended treatment.

Our findings have important implications for China and other countries in which the healthcare delivery system is much weaker in rural areas than in urban areas. Rural dwellers are older, more impoverished, and live in sparsely populated areas where medical resources are much less concentrated [17, 46]. However, as it is the standard and preferred treatment for AMI, PCI should be equally accessible in rural and urban hospitals. Our study highlights the need for policymakers to prioritize policy efforts to improve access to PCI procedures in rural areas (such as equipping rural hospitals with qualified physicians and infrastructure and improving the reimbursement ratio of PCI for a patient having the New Rural Cooperative Medical Scheme) to achieve equitable treatment and outcome for AMI patients in rural and urban hospitals [47]. Our results may also be relevant to other regions in the world as a spate of studies has shown the rural-urban disparity in in-hospital mortality rates and adoption rates of PCI in other countries [48–51]. The coherent evidence on the exposure-outcome and exposure-mediator associations from other countries enhances the plausibility and potential generalizability of PCI mediating the rural-urban disparity in AMI in-hospital mortality, suggesting the need to strength policy efforts to eliminate rural-urban disparity in health outcomes by ameliorating the access to guideline recommended treatments such as primary PCI.

This study includes a large and recent sample of diverse in patient characteristics and is highly representative of the province-wide hospitalized population. In contrast to previous studies that modeled the association between hospital rurality or PCI on in-hospital mortality, we further applied mediation analyses to quantitatively estimate how the effect of hospital rurality translates into in-hospital mortality through PCI procedures and identify actionable solutions to address inequalities.

Compared to previous studies that used a national but smaller sample of patients collected in 2011 [13, 52, 53], our study includes a lower percentage of STEMI patients (53.2% versus 74.2%) but a higher percentage of PCI rates (37.8% versus 28.1%). The lower portion of STEMI patients is likely to reflect the difference in the underlying population and potential misclassification in ICD-10-CM codes; a higher rate of PCI may suggest an increasing trend toward PCI use in Chinese hospitals. Other studies also investigated the mediating effect of PCI on AMI mortality associated with Finland and Norway’s socioeconomic status, but they reported little to no extent of mediating effect [54].

Compared to previous randomized controlled trials that evaluated PCI’s effect on AMI patients [25], the association between PCI and in-hospital mortality (OR: 0.20, 95% CI: 0.16–0.24) seemed too protective to be accurate. This possibly reflects the indication of PCI, which typically includes younger and healthier patients and those who received care in time, and these patients who met the indication of PCI had much lower overall in-hospital mortality.

This study has several limitations. Since the study is based on the discharge data for hospitalized patients, other essential variables, including time to treatment, smoking status, and medications such as aspirin and beta-blockers, are not available. Since the treatment rurality, mediator PCI, and outcome in-hospital mortality were all binary variables, sensitivity analyses for sequential ignorability, which is a crucial assumption for mediation analysis, cannot be performed due to modeling limitations [43]. Our data were collected from Shanxi, so the results may not generalize to patients hospitalized in other provinces. All clinical diseases and comorbidities were ascertained using ICD-10 codes, which may be subject to misclassification bias. We did not assess the rurality of the patients. Therefore, we cannot verify the outcomes of rural patients treated in urban hospitals nor the outcomes of urban patients who sought care in rural hospitals.

### Conclusion

In summary, our results suggest that PCI mediates a large proportion of the association of hospital rurality with the outcomes of AMI patients in China. The results highlight the need to improve the essential infrastructure of rural hospitals, which can help further address the inequalities of treatments and outcomes in rural and urban hospitals.

## Data Availability

The data cannot be shared due to institutional policies. The code can be shared upon request to MC, miao.cai@outlook.com.
