# Supplementary material for: The Chasm in Percutaneous Coronary Intervention and In-Hospital Mortality Rates Among Acute Myocardial Infarction Patients in Rural and Urban Hospitals in China: A Mediation Analysis
Source: Int J Public Health. 2022 Jul 7;67:1604846. doi: 10.3389/ijph.2022.1604846 (PMC9302370; doi:10.3389/ijph.2022.1604846)
Supplement: Supplementary file 1 [file DataSheet1.doc]

**Supplemental materials**

**The Chasm in Percutaneous Coronary Intervention and In-hospital Mortality Rates among Acute Myocardial Infarction Patients in Rural and Urban Hospitals in China: A Mediation Analysis**

| **Tables** | **Page** |
| --- | --- |
| **Supplemental Table 1:** Rural-urban area classification codes and associated meanings (Shanxi, China. 2013-2017). | 1 |
| **Supplemental Table 2:** Association between hospital rurality and non-recovery for acute myocardial infarction using different adjustment methods (Shanxi, China. 2013-2017). | 2 |
| **Supplemental Table 3:** Association between hospital rurality and in-hospital mortality for acute myocardial infarction using different adjustment methods in different subtypes of acute myocardial infarction (Shanxi, China. 2013-2017). | 3 |
| **Supplemental Table 4:** Association between hospital rurality and non-recovery for acute myocardial infarction using different adjustment methods in different subtypes of acute myocardial infarction (Shanxi, China. 2013-2017). | 4 |
| **Supplemental Table 5:** Percutaneous coronary intervention mediating the effect of hospital rurality on non-recovery in overall sample and subgroups of acute myocardial infarction patients (Shanxi, China. 2013-2017). | 5 |

| **Supplemental Table 1:** Rural-urban area classification codes and associated meanings (Shanxi, China. 2013-2017). | | |
| --- | --- | --- |
| **Classification code** | **Meaning** | **Rural urban classification** |
| 111 | Main urban district | Urban |
| 121 | Center areas of a town |
| 112 | Urban-town fringe | Rural |
| 122 | Town-village fringe |
| 123 | Special areas of a town |
| 210 | Center areas of a village |
| 220 | Villages |

| **Supplemental Table 2:** Association between hospital rurality and non-recovery for acute myocardial infarction using different adjustment methods (Shanxi, China. 2013-2017). | | | | |
| --- | --- | --- | --- | --- |
| **Variables** | **Unadjusted** | **Minimally sufficient set** | **Full** | **IPW** |
| Rural | 1.61 (1.49, 1.73) | 1.46 (1.35, 1.59) | 1.11 (1.02, 1.20) | 1.12 (1.07, 1.18) |
| Age in 10 years |  | 1.70 (1.65, 1.76) | 1.54 (1.49, 1.59) | 1.50 (1.47, 1.53) |
| Female |  | 1.41 (1.31, 1.52) | 1.35 (1.26, 1.46) | 1.31 (1.24, 1.38) |
| Marital status (reference = married) | | | | |
| Unmarried |  | 0.97 (0.73, 1.26) | 0.96 (0.72, 1.25) | 0.97 (0.81, 1.17) |
| Widowed |  | 1.27 (1.11, 1.46) | 1.18 (1.03, 1.35) | 1.28 (1.16, 1.40) |
| Divorced |  | 0.88 (0.69, 1.10) | 0.84 (0.66, 1.06) | 0.69 (0.56, 0.84) |
| Other |  | 1.35 (1.04, 1.73) | 1.22 (0.94, 1.56) | 1.20 (1.00, 1.43) |
| Occupation (reference = public institution) | | | | |
| Private institution |  | 1.14 (0.92, 1.42) | 1.24 (1.00, 1.55) | 1.21 (1.05, 1.41) |
| Farmer |  | 1.29 (1.06, 1.57) | 1.20 (0.99, 1.46) | 1.27 (1.11, 1.45) |
| Jobless |  | 1.35 (1.07, 1.72) | 1.26 (1.00, 1.61) | 1.39 (1.18, 1.64) |
| Retired |  | 1.32 (1.08, 1.62) | 1.30 (1.07, 1.60) | 1.27 (1.11, 1.46) |
| Other |  | 1.17 (0.94, 1.45) | 1.01 (0.81, 1.26) | 1.08 (0.94, 1.26) |
| Severity upon admission (reference = normal) | | | | |
| Emergent |  | 0.96 (0.88, 1.05) | 0.99 (0.90, 1.08) | 0.98 (0.92, 1.04) |
| Dangerous |  | 1.93 (1.79, 2.08) | 2.03 (1.88, 2.19) | 1.94 (1.83, 2.05) |
| Hypertension |  | 0.79 (0.74, 0.85) | 0.81 (0.75, 0.86) | 0.79 (0.75, 0.83) |
| Diabetes mellitus |  | 1.10 (1.02, 1.20) | 1.13 (1.04, 1.22) | 1.08 (1.02, 1.14) |
| Renal disease |  | 2.38 (2.02, 2.80) | 2.00 (1.69, 2.34) | 2.50 (2.23, 2.79) |
| AMI type (reference = STEMI) | | | | |
| Non-STEMI |  | 0.66 (0.60, 0.73) | 0.56 (0.51, 0.62) | 0.63 (0.59, 0.67) |
| Non-specified |  | 1.25 (1.16, 1.35) | 1.14 (1.06, 1.23) | 1.10 (1.04, 1.16) |
| PCI |  |  | 0.15 (0.13, 0.17) | 0.19 (0.17, 0.20) |
| Effect estimates are presented as odds ratios with 95% confidence intervals. The minimally sufficient set is selected based on the directed acyclic graph in Figure 1. IPW: inverse probability weighting, AMI: acute myocardial infarction; STEMI: ST-elevation myocardial infarction; PCI: Percutaneous coronary intervention. | | | | |

| **Supplemental Table 3:** Association between hospital rurality and in-hospital mortality for acute myocardial infarction using different adjustment methods in different subtypes of acute myocardial infarction (Shanxi, China. 2013-2017). | | | | | | | | | |
| --- | --- | --- | --- | --- | --- | --- | --- | --- | --- |
| **AMI subtypes** | **STEMI (*n*=43,944)** | | | **Non-STEMI (*n*=18,797)** | | | **Non-specified (*n*=19,936)** | | |
| **Variables** | **MSS** | **Full** | **IPW** | **MSS** | **Full** | **IPW** | **MSS** | **Full** | **IPW** |
| Rural | 1.47 (1.22, 1.76) | 1.15 (0.96, 1.39) | 1.11 (1.00, 1.24) | 1.10 (0.62, 1.83) | 0.96 (0.54, 1.59) | 1.12 (0.93, 1.35) | 1.08 (0.82, 1.42) | 0.84 (0.64, 1.10) | 0.82 (0.62, 1.07) |
| Age in 10 years | 1.72 (1.60, 1.85) | 1.57 (1.46, 1.69) | 1.62 (1.54, 1.70) | 2.07 (1.80, 2.39) | 1.84 (1.59, 2.12) | 2.09 (1.89, 2.32) | 1.74 (1.59, 1.91) | 1.56 (1.43, 1.71) | 1.50 (1.40, 1.60) |
| Female | 1.42 (1.19, 1.69) | 1.38 (1.15, 1.64) | 1.45 (1.29, 1.64) | 1.17 (0.87, 1.56) | 1.13 (0.85, 1.51) | 0.75 (0.61, 0.93) | 1.42 (1.15, 1.75) | 1.36 (1.10, 1.67) | 1.38 (1.18, 1.61) |
| Marital status (reference = married) | | | | | | | | | |
| Unmarried | 0.97 (0.48, 1.73) | 0.98 (0.48, 1.75) | 1.02 (0.65, 1.52) | 0.33 (0.02, 1.52) | 0.32 (0.02, 1.48) | 0.15 (0.01, 0.66) | 0.82 (0.29, 1.82) | 0.79 (0.28, 1.76) | 0.72 (0.34, 1.35) |
| Widowed | 1.12 (0.81, 1.51) | 1.05 (0.76, 1.42) | 1.11 (0.89, 1.37) | 0.75 (0.42, 1.24) | 0.71 (0.40, 1.17) | 1.19 (0.85, 1.63) | 1.24 (0.83, 1.79) | 1.12 (0.75, 1.62) | 1.40 (1.04, 1.83) |
| Divorced | 1.02 (0.58, 1.64) | 0.97 (0.56, 1.58) | 0.61 (0.37, 0.95) | 0.76 (0.31, 1.55) | 0.74 (0.31, 1.52) | 0.50 (0.21, 1.02) | 1.56 (0.93, 2.49) | 1.56 (0.93, 2.50) | 1.25 (0.79, 1.90) |
| Other | 1.72 (0.95, 2.88) | 1.53 (0.84, 2.57) | 1.78 (1.22, 2.50) | 0.58 (0.09, 1.88) | 0.52 (0.08, 1.69) | 0.18 (0.03, 0.55) | 1.51 (0.71, 2.85) | 1.37 (0.64, 2.59) | 1.22 (0.71, 1.96) |
| Occupation (reference = public institution) | | | | | | | | | |
| Private institution | 1.20 (0.77, 1.96) | 1.34 (0.85, 2.18) | 1.41 (1.03, 1.97) | 0.54 (0.25, 1.29) | 0.53 (0.24, 1.27) | 0.96 (0.63, 1.49) | 0.70 (0.39, 1.26) | 0.75 (0.42, 1.36) | 1.58 (1.03, 2.51) |
| Farmer | 0.86 (0.57, 1.35) | 0.80 (0.53, 1.26) | 0.93 (0.70, 1.26) | 0.59 (0.30, 1.32) | 0.56 (0.28, 1.24) | 0.37 (0.24, 0.57) | 0.80 (0.50, 1.34) | 0.77 (0.48, 1.30) | 1.16 (0.79, 1.79) |
| Jobless | 1.11 (0.65, 1.93) | 1.06 (0.62, 1.84) | 0.89 (0.60, 1.33) | 0.42 (0.15, 1.15) | 0.38 (0.14, 1.04) | 0.13 (0.06, 0.28) | 0.80 (0.41, 1.54) | 0.77 (0.40, 1.49) | 1.46 (0.89, 2.42) |
| Retired | 1.45 (0.95, 2.30) | 1.47 (0.97, 2.34) | 1.58 (1.18, 2.17) | 1.35 (0.70, 2.93) | 1.27 (0.66, 2.76) | 0.56 (0.37, 0.86) | 1.62 (1.02, 2.73) | 1.63 (1.02, 2.75) | 1.93 (1.30, 2.99) |
| Other | 0.92 (0.58, 1.51) | 0.80 (0.50, 1.32) | 0.84 (0.60, 1.17) | 0.71 (0.32, 1.67) | 0.66 (0.30, 1.56) | 0.43 (0.27, 0.70) | 0.74 (0.42, 1.35) | 0.64 (0.36, 1.18) | 0.97 (0.62, 1.58) |
| Severity upon admission (reference = normal) | | | | | | | | | |
| Emergent | 0.82 (0.67, 1.01) | 0.85 (0.69, 1.04) | 1.01 (0.88, 1.15) | 0.57 (0.38, 0.84) | 0.57 (0.38, 0.83) | 0.44 (0.32, 0.60) | 1.15 (0.88, 1.48) | 1.19 (0.92, 1.54) | 1.16 (0.96, 1.41) |
| Dangerous | 1.76 (1.48, 2.09) | 1.86 (1.56, 2.21) | 1.59 (1.40, 1.79) | 2.60 (1.96, 3.44) | 2.56 (1.93, 3.39) | 2.84 (2.32, 3.47) | 2.09 (1.70, 2.58) | 2.20 (1.78, 2.71) | 1.97 (1.68, 2.31) |
| Hypertension | 0.87 (0.74, 1.02) | 0.89 (0.76, 1.03) | 0.73 (0.65, 0.81) | 0.70 (0.53, 0.91) | 0.70 (0.54, 0.91) | 0.76 (0.63, 0.92) | 0.73 (0.60, 0.88) | 0.72 (0.60, 0.88) | 0.58 (0.50, 0.67) |
| Diabetes mellitus | 0.96 (0.78, 1.16) | 0.97 (0.80, 1.18) | 0.81 (0.70, 0.94) | 1.06 (0.78, 1.41) | 1.06 (0.78, 1.43) | 1.27 (1.04, 1.56) | 1.38 (1.11, 1.71) | 1.41 (1.13, 1.74) | 1.45 (1.24, 1.71) |
| Renal disease | 1.98 (1.31, 2.87) | 1.68 (1.11, 2.44) | 1.99 (1.52, 2.55) | 2.13 (1.30, 3.35) | 1.93 (1.18, 3.03) | 5.63 (4.44, 7.10) | 2.67 (1.72, 4.01) | 2.22 (1.43, 3.33) | 4.01 (3.00, 5.28) |
| PCI |  | 0.23 (0.18, 0.29) | 0.25 (0.21, 0.30) |  | 0.19 (0.10, 0.33) | 0.24 (0.15, 0.37) |  | 0.15 (0.10, 0.22) | 0.14 (0.09, 0.19) |
| Effect estimates are presented as odds ratios with 95% confidence intervals. MSS: minimally sufficient set, which is selected based on the directed acyclic graph in Figure 1. IPW: inverse probability weighting; AMI: acute myocardial infarction; STEMI: ST-elevation myocardial infarction; PCI: Percutaneous coronary intervention. | | | | | | | | | |

| **Supplemental Table 4:** Association between hospital rurality and non-recovery for acute myocardial infarction using different adjustment methods in different subtypes of acute myocardial infarction (Shanxi, China. 2013-2017). | | | | | | | | | |
| --- | --- | --- | --- | --- | --- | --- | --- | --- | --- |
| **AMI subtypes** | **STEMI (*n*=43,944)** | | | **Non-STEMI (*n*=18,797)** | | | **Non-specified (*n*=19,936)** | | |
| **Variables** | **MSS** | **Full** | **IPW** | **MSS** | **Full** | **IPW** | **MSS** | **Full** | **IPW** |
| Rural | 1.49 (1.34, 1.66) | 1.13 (1.01, 1.26) | 1.10 (1.03, 1.17) | 1.69 (1.29, 2.19) | 1.41 (1.07, 1.82) | 1.37 (1.23, 1.52) | 1.33 (1.15, 1.53) | 0.99 (0.86, 1.14) | 0.95 (0.87, 1.03) |
| Age in 10 years | 1.68 (1.61, 1.76) | 1.52 (1.46, 1.59) | 1.48 (1.43, 1.52) | 1.73 (1.60, 1.88) | 1.53 (1.42, 1.66) | 1.59 (1.50, 1.67) | 1.71 (1.61, 1.81) | 1.55 (1.47, 1.64) | 1.45 (1.40, 1.51) |
| Female | 1.44 (1.30, 1.60) | 1.39 (1.25, 1.54) | 1.40 (1.30, 1.50) | 1.20 (1.00, 1.43) | 1.15 (0.96, 1.37) | 0.96 (0.85, 1.08) | 1.51 (1.32, 1.72) | 1.44 (1.26, 1.64) | 1.45 (1.32, 1.59) |
| Marital status (reference = married) | | | | | | | | | |
| Unmarried | 1.03 (0.70, 1.46) | 1.04 (0.71, 1.47) | 1.22 (0.96, 1.51) | 0.76 (0.32, 1.51) | 0.74 (0.31, 1.48) | 0.72 (0.45, 1.10) | 1.00 (0.60, 1.58) | 0.96 (0.57, 1.51) | 0.95 (0.67, 1.31) |
| Widowed | 1.38 (1.15, 1.65) | 1.29 (1.07, 1.55) | 1.37 (1.20, 1.55) | 1.05 (0.76, 1.42) | 0.97 (0.71, 1.32) | 1.50 (1.24, 1.80) | 1.27 (0.97, 1.63) | 1.15 (0.88, 1.48) | 1.22 (1.01, 1.47) |
| Divorced | 0.87 (0.60, 1.21) | 0.82 (0.57, 1.15) | 0.70 (0.52, 0.91) | 0.52 (0.26, 0.92) | 0.50 (0.25, 0.88) | 0.31 (0.16, 0.54) | 1.15 (0.77, 1.66) | 1.14 (0.76, 1.66) | 0.79 (0.56, 1.08) |
| Other | 1.50 (1.03, 2.11) | 1.32 (0.91, 1.87) | 1.55 (1.22, 1.95) | 0.83 (0.37, 1.61) | 0.74 (0.33, 1.43) | 0.24 (0.11, 0.44) | 1.44 (0.93, 2.15) | 1.32 (0.85, 1.97) | 1.25 (0.93, 1.66) |
| Occupation (reference = public institution) | | | | | | | | | |
| Private institution | 1.34 (0.99, 1.85) | 1.51 (1.11, 2.09) | 1.70 (1.37, 2.13) | 1.34 (0.80, 2.34) | 1.32 (0.79, 2.31) | 0.83 (0.64, 1.08) | 0.82 (0.57, 1.20) | 0.88 (0.61, 1.29) | 1.04 (0.82, 1.33) |
| Farmer | 1.47 (1.12, 1.97) | 1.34 (1.02, 1.80) | 1.75 (1.44, 2.15) | 1.29 (0.81, 2.20) | 1.21 (0.76, 2.07) | 0.61 (0.48, 0.78) | 1.08 (0.80, 1.50) | 1.04 (0.76, 1.45) | 1.11 (0.91, 1.38) |
| Jobless | 1.77 (1.26, 2.50) | 1.67 (1.19, 2.38) | 1.92 (1.50, 2.46) | 1.30 (0.73, 2.39) | 1.16 (0.65, 2.14) | 0.46 (0.33, 0.64) | 0.97 (0.64, 1.48) | 0.92 (0.61, 1.41) | 1.02 (0.77, 1.35) |
| Retired | 1.39 (1.04, 1.88) | 1.40 (1.05, 1.91) | 1.91 (1.56, 2.37) | 1.49 (0.93, 2.55) | 1.40 (0.87, 2.39) | 0.55 (0.43, 0.72) | 1.16 (0.84, 1.62) | 1.15 (0.83, 1.61) | 1.15 (0.93, 1.44) |
| Other | 1.29 (0.95, 1.77) | 1.09 (0.80, 1.51) | 1.43 (1.16, 1.79) | 1.20 (0.71, 2.13) | 1.11 (0.66, 1.97) | 0.58 (0.44, 0.77) | 1.04 (0.73, 1.51) | 0.89 (0.62, 1.30) | 1.03 (0.81, 1.32) |
| Severity upon admission (reference = normal) | | | | | | | | | |
| Emergent | 0.93 (0.83, 1.05) | 0.97 (0.86, 1.09) | 1.01 (0.94, 1.09) | 0.96 (0.78, 1.18) | 0.95 (0.77, 1.16) | 0.82 (0.71, 0.94) | 0.99 (0.84, 1.17) | 1.03 (0.87, 1.22) | 0.94 (0.84, 1.06) |
| Dangerous | 1.72 (1.54, 1.91) | 1.84 (1.65, 2.05) | 1.62 (1.50, 1.75) | 2.47 (2.06, 2.97) | 2.44 (2.03, 2.93) | 1.94 (1.70, 2.21) | 2.05 (1.79, 2.35) | 2.14 (1.86, 2.45) | 2.00 (1.82, 2.19) |
| Hypertension | 0.90 (0.82, 0.99) | 0.92 (0.83, 1.01) | 0.85 (0.80, 0.91) | 0.70 (0.59, 0.82) | 0.70 (0.60, 0.83) | 0.78 (0.70, 0.87) | 0.70 (0.61, 0.79) | 0.70 (0.61, 0.79) | 0.67 (0.62, 0.73) |
| Diabetes mellitus | 1.05 (0.93, 1.18) | 1.08 (0.96, 1.21) | 1.05 (0.97, 1.14) | 0.99 (0.82, 1.18) | 1.00 (0.82, 1.20) | 1.01 (0.89, 1.14) | 1.28 (1.11, 1.48) | 1.31 (1.14, 1.52) | 1.27 (1.15, 1.40) |
| Renal disease | 2.23 (1.73, 2.83) | 1.85 (1.43, 2.35) | 1.91 (1.60, 2.26) | 2.70 (2.00, 3.61) | 2.37 (1.75, 3.16) | 4.29 (3.63, 5.06) | 2.28 (1.64, 3.12) | 1.87 (1.34, 2.55) | 2.36 (1.87, 2.95) |
| PCI |  | 0.16 (0.14, 0.19) | 0.19 (0.17, 0.21) |  | 0.13 (0.09, 0.19) | 0.14 (0.10, 0.18) |  | 0.13 (0.09, 0.16) | 0.12 (0.09, 0.15) |
| Effect estimates are presented as odds ratios with 95% confidence intervals. MSS: minimally sufficient set, which is selected based on the directed acyclic graph in Figure 1. IPW: inverse probability weighting; AMI: acute myocardial infarction; STEMI: ST-elevation myocardial infarction; PCI: Percutaneous coronary intervention. | | | | | | | | | |

| **Supplemental Table 5:** Percutaneous coronary intervention mediating the effect of hospital rurality on non-recovery in overall sample and subgroups of acute myocardial infarction patients (Shanxi, China. 2013-2017). | | | | | | | | |
| --- | --- | --- | --- | --- | --- | --- | --- | --- |
| **Sample** | **Overall (N=82,677)** | | **STEMI (N=43,944)** | | **Non-STEMI (N=18,797)** | | **Non-specified (N=19,936)** | |
| **Odd ratio estimates** | **Estimate (95% CI)** | **P-value** | **Estimate (95% CI)** | **P-value** | **Estimate (95% CI)** | **P-value** | **Estimate (95% CI)** | **P-value** |
| Total effect | 1.019 (1.015, 1.02) | <0.001 | 1.02 (1.015, 1.03) | <0.001 | 1.021 (1.009, 1.03) | <0.001 | 1.019 (1.01, 1.03) | <0.001 |
| Average causal mediation  effect (ACME) | 1.014 (1.013, 1.01) | <0.001 | 1.015 (1.014, 1.02) | <0.001 | 1.007 (1.006, 1.01) | <0.001 | 1.02 (1.017, 1.02) | <0.001 |
| Average direct effect (ADE) | 1.005 (1.001, 1.01) | 0.014 | 1.005 (1, 1.01) | 0.031 | 1.013 (1.003, 1.03) | 0.011 | 0.999 (0.991, 1.01) | 0.86 |
| **Proportion estimates** | | | | | | | | |
| Proportion mediated | 74.6%  (63.0%, 97.0%) | <0.001 | 72.8%  (60.3%, 101.0%) | <0.001 | 35.4%  (26.9%, 100.0%) | <0.001 | 103.9%  (78.1%, 232.0%) | <0.001 |

STEMI: ST-elevation myocardial infarction; PCI: percutaneous coronary intervention; CI: confidence interval.
